# Toward optimizing diversifying base editors for high-throughput mutational scanning studies

**DOI:** 10.1093/nar/gkaf620

**Published:** 2025-07-04

**Authors:** Carley I Schwartz, Nathan S Abell, Amy Li, Aradhana, Josh Tycko, Alisa Truong, Stephen B Montgomery, Gaelen T Hess

**Affiliations:** Department of Biomolecular Chemistry, School of Medicine and Public Health, University of Wisconsin-Madison, Madison, WI 53705, United States; Center for Human Genomics and Precision Medicine, School of Medicine and Public Health, University of Wisconsin-Madison, Madison, WI 53705, United States; Department of Genetics, Stanford University, Stanford, CA 94305, United States; Department of Genetics, Stanford University, Stanford, CA 94305, United States; Department of Genetics, Stanford University, Stanford, CA 94305, United States; Department of Genetics, Stanford University, Stanford, CA 94305, United States; Department of Genetics, Stanford University, Stanford, CA 94305, United States; Department of Genetics, Stanford University, Stanford, CA 94305, United States; Department of Pathology, Stanford University, Stanford, CA 94305, United States; Department of Biomolecular Chemistry, School of Medicine and Public Health, University of Wisconsin-Madison, Madison, WI 53705, United States; Center for Human Genomics and Precision Medicine, School of Medicine and Public Health, University of Wisconsin-Madison, Madison, WI 53705, United States

## Abstract

Base editors, including diversifying base editors that create C>N mutations, are potent tools for systematically installing point mutations in mammalian genomes and studying their effect on cellular function. Numerous base editor options are available for such studies, but little information exists on how the composition of the editor (deaminase, recruitment method, and fusion architecture) affects editing. To address this knowledge gap, the effect of various design features, such as deaminase recruitment and delivery method (electroporation or lentiviral transduction), on editing was assessed across ∼200 synthetic target sites. The direct fusion of a hyperactive variant of activation-induced cytidine deaminase to the N-terminus of dCas9 (DivA-BE) produced the highest editing efficiency, ∼4-fold better than the previous CRISPR-X method. Additionally, DivA-BE mutagenized the DNA strand that anneals to the targeting sgRNA (target strand) to create complementary C>N mutations, which were absent when the deaminase was fused to the C-terminus of dCas9. Based on these studies that comprehensively analyze the editing patterns of several popular base editors, DivA-BE editors efficiently diversified their target sites, albeit with increased indel frequencies. Overall, the improved editing efficiency makes the DivA-BE editors ideal for discovering functional variants in mutational scanning assays.

## Graphical Abstract

**Graphical Abstract:**
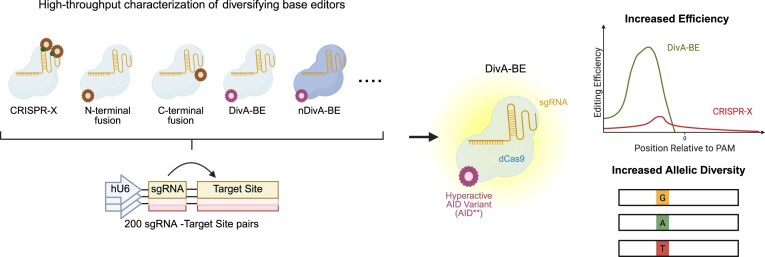

## Introduction

Advances in mutational scanning have enabled in-depth, systematic mapping of genetic variation to phenotypes. Through these high-throughput studies, analysis of functional effects at the single-nucleotide scale has allowed researchers to progress in understanding variations in protein interactions, establishing quantitative evolutionary models, and probing the effects of single-nucleotide variants (SNVs) in disease-associated genes [1–3]. However, the benefits of mutational scanning can only extend as far as mutation-installing technologies allow it.

Base editors are potent genome editors that can introduce programmable mutations, most commonly C>T or A>G, in endogenous genomic DNA, allowing the effect of individual base substitutions in cell or animal models to be studied. As these editors do not generate toxic DNA double-strand breaks, they reduce the introduction of insertions/deletions (indels) that can result in unwanted gene knockouts. In addition to these programmable base editors, diversifying cytidine base editors (DCBEs) that produce C>N edits are powerful tools for annotating thousands of mutations in high-throughput assays. These diversifying base editors hold an advantage over C>T editors as they produce more substitutions that can be interrogated for phenotypic effects. For diversifying and programmable base editors, characterization of their properties (efficiency, targeting window, the spectrum of substitutions, etc.) is necessary for selecting the optimal editor for high-throughput editing.

Given the number of base editor options available for these scanning mutagenesis studies, it is critical to define the properties of base editors to determine the editor most suited for the intended application. Understanding how the base editor configuration (e.g. method of recruiting or fusing the deaminase to the Cas9 protein) affects editing properties would facilitate the creation of editors better suited for their specific studies. Defining the properties of editors would benefit from the parallel assessment of the editor’s activity across many target sites, as underlying sequence variation can influence editing outcomes. While such studies have profiled programmable editors [4, 5], to date, studies of DCBEs have focused on editing for a limited number of target sequences [6–8].

Here, a comprehensive approach was established and used to assess the impact of several design features on the editing outcomes of DCBEs for ∼200 sgRNA-target site pairs simultaneously. We determined the editing efficiency, targeting window, and base substitution spectrum for base editors expressed transiently or constitutively. The recruitment method for a hyperactive variant of the activation-induced cytidine deaminase (AID) affected the editing efficiency and targeting window. These studies described an improved diversifying base editor, DivA-BE (Diversifying AID fusion Base Editor), consisting of a hyperactive AID variant fused to the N-terminus of catalytically inactive Cas9 (dCas9) with improved editing efficiency and a reduced targeting window compared to the previously established CRISPR-X editor [6]. We found that deamination of the target strand that anneals to the sgRNA produced additional diversification for DivA-BE, but these edits were depleted for editors with the AID variant fused to the C-terminus of dCas9 and editors using the rat APOBEC1 (rAPOBEC1) deaminase. Our analyses revealed that fusing the hyperactive AID variant to nCas9 (nDivA-BE) produced the most diversification at the target sites but also exhibited the highest frequency of indels, which may be a drawback in assays testing the phenotypic effect of mutations in coding regions. These and other data described in this report should serve as an important resource for maximizing the effectiveness of future high-throughput editing experiments.

## Materials and methods

### Design of sgRNA-target site library

To design the sgRNA library, we selected 2000 20-bp sgRNA sequences from non-targeting controls generated for a human genome-wide CRISPR knockout library [9]. The synthetic target site was 104 bp long, centered on an NGG PAM compatible with editing by *Streptococcus pyogenes* Cas9 (Cas9). The N in the NGG and the surrounding sequence beyond the sgRNA were randomly generated with a 52% GC bias to represent the content of the human genome. For half of the sites, the sequence was reverse-complemented. sgRNAs and target sites containing restriction enzyme sites (BsmBI/Esp3I, BsaI, and BlpI) required for cloning were removed, and 200 sgRNA-target site pairs were selected randomly. For 10 sgRNA-target site pairs, the sgRNA sequence in the target site was scrambled to produce negative control sgRNA-target site pairs. A linker sequence (5′-GTTTGGAGACGGGATACCGTCTCTGATC-3′) containing two BsmBI sites was placed after the sgRNA protospacer sequence and before the target site. This linker sequence was used to insert the sgRNA stem-loop during cloning. The oligonucleotides were extended on both ends to add sequences to enable polymerase chain reaction (PCR) amplification of the oligonucleotides and add restriction sites compatible with cloning at the 5′ (BsaI) and 3′ (BlpI) sites, respectively. A complete list of the designed oligonucleotides is in Supplementary Table S1. These designed oligonucleotides were synthesized by Agilent.

### Construction of paired sgRNA-target site library

The sequences of primers used to generate the sgRNA-target site library are listed in Supplementary Table S2. The sgRNA-target site oligonucleotides were cloned into pGH643. This vector contained a human U6 promoter expressing an sgRNA upstream of an Ef1ɑ promoter driving the expression of puromycin resistance and mCherry used for downstream selection and monitoring infection percentage. A 100 μl restriction digest of pGH643 was incubated overnight at 37°C [10 μg pGH643, 4.5 μl Esp3I (NEB, R0734L), 4.5 μl BlpI (NEB, R0585L), and 10 μl of CutSmart Buffer (NEB)]. The digest reaction was run on a 1% TAE gel, and the band was excised and purified with the QiaQuick Gel Extraction Kit (Qiagen, 28706). The oligonucleotide library insert was produced using 4 × 100 μl PCR reactions. Each reaction contained 1.5 ng (synthesized oligonucleotide template), 1 μl of Herculase II polymerase (Agilent, 600679), 2 μl 10 mM dNTPs (Roche 11969064001), 2 μl 100 μM forward primer, 2 μl 100 μM reverse primer, 2 μl DMSO, and 20 μl of 5× Herculase buffer. The thermocycling conditions were 98°C for 3 min, 11 cycles of 98°C for 30 sec, 55°C for 30 sec, and 72°C for 30 sec, followed by a final extension of 72°C for 3 min. The reactions were pooled and 5 μl was analyzed on a 2% TBE agarose gel to confirm the correct amplicon size. The remaining PCR reaction was purified using the QiaQuick PCR Purification Kit (Qiagen, 28104) and eluted in EB buffer. The purified PCR was digested overnight at 37°C in a 120 μl reaction [5 μl BsaI-HF (NEB, R3733L), 5 μl BlpI, and 12 μl of CutSmart Buffer]. The digested insert was analyzed on a 3% TBE agarose gel and bands were extracted and purified. A 100 μl ligation reaction was set up as follows: 2 μg digested pGH643, 360 ng digested insert (∼12:1 ratio), 5 μl of T4 Ligase (NEB, M0202L), and 10 μl T4 Ligase Buffer (NEB, B0202S). The reaction was incubated for 16 h at 16°C followed by 70°C for 15 min. The ligation reaction was purified and concentrated to >100 ng/μl using MinElute PCR Purification Kit (Qiagen, 28006). 200 ng of purified ligation was electroporated into 60 μl of electrocompetent Lucigen Endura cells (Biosearch Technologies 60242-1) according to manufacturer’s instructions. Cells were rescued in 4 ml of Lucigen recovery media and grown for 1 h at 37°C. The library was plated on 8 × 1 sq ft. LB agar (Fisher, DF0445076) plates with carbenicillin (Fisher, 50213248, 100 μg/ml). Two smaller plates were used to titer the number of transformed colonies and guarantee the library had >100× coverage per sgRNA-target site pair. The 1 sq ft. LB agar plates were scraped to collect colonies, and DNA was extracted using the HiSpeed Plasmid Maxi Kit (Qiagen, 12663).

A second round of cloning was performed to introduce the stem-loop between the sgRNA protospacer and the target site. The no stem-loop plasmid library was digested for 8 h at 55°C [10 μg plasmid library, 9 μl BsmBI (NEB, R0739L), and 10 μl of Buffer 3.1 (NEB, B6003S)]. The resulting digest was analyzed on a 1% TAE agarose gel and purified. The stem-loop insert was prepared by 4 × 100 μl PCR reactions consisting of 1 ng of pGH643 (stem-loop template), 1 μl of Herculase II polymerase, 2 μl 10 mM dNTPs, 2 μl 100 μM oGH915, 2 μl 100 μM oGH1001, 2 μl DMSO, and 20 μl of 5× Herculase buffer. oGH1001 included seven degenerate nucleotides that serve as a unique molecular identifier (UMI). This UMI was inserted between the end of the stem-loop and the start of the target site. The thermocycling conditions were 98°C for 3 min, 15 cycles of 98°C for 30 sec, 55°C for 30 sec, and 72°C for 30 sec, followed by a final extension of 72°C for 3 min. The fragment was analyzed on a 2% TBE agarose gel, followed by gel extraction and purification. The purified PCR product was digested for 8 h at 55°C in a 120 μl reaction (9 μl BsmBI and 12 μl Buffer 3.1), followed by PCR purification. A 100 μl ligation reaction consisting of 2 μg of digested library, 280 ng digested stem-loop (∼10:1 ratio), 5 μl T4 Ligase, and 10 μl T4 Ligase Buffer was incubated at 16°C for 16 h. The reaction cleanup, electroporation, and DNA extraction process were repeated as described in the first round of cloning.

### Construction of base editor expression vectors

A complete list of plasmids and their sequences is listed in Supplementary Table S2. Expression constructs for MS2-AID variant fusions (pGH335, pGH183) and dCas9 (pGH125) were previously described [6]. pGH335 and pGH183 contained an Ef1ɑ promoter driving the expression of the MS2 coat protein fused to an AID variant. These vectors were upstream of a T2A-hygromycin resistance (HygroR) cassette. Both vectors were digested with BsrGI-HF (NEB, R3575L) and EcoRI-HF (NEB, R3101L) to remove the HygroR. A PCR-amplified T2A-GFP cassette was inserted in place of HygroR by Gibson Assembly. pGH125 contained an Ef1ɑ promoter driving the expression of dCas9 followed by a T2A-blasticidin resistance (BlastR) gene. A fluorescent dCas9-expression vector (pGH483) was generated by digesting pGH125 with BamHI-HF (NEB, R3136L) and EcoRI-HF to remove T2A-BlastR. A T2A-tagBFP cassette was amplified by PCR and inserted into the digested pGH125 via Gibson Assembly. pGH125 or pGH483 were digested with BsiWI-HF (NEB, R3553L) and AfeI (NEB, R0652S) to remove dCas9, and genome editors were inserted into the vector via Gibson Assembly. pGH896, which expressed DivA-BE, was digested with BamHI-HF and EcoRI-HF to remove the T2A-tagBFP, and T2A-GFP was inserted in its place to generate pGH941.

### Cell culture

K562 cells, a chronic myeloid leukemia model, were cultured in RPMI 1640 media (Gibco, 11875119) supplemented with 10% fetal bovine serum (FBS) (Life Technologies, 10437028), 2 mM l-glutamine (VWR, 16777-162), and 1% penicillin/streptomycin (VWR, 16777-164). HEK293T cells were grown in Dulbecco’s modified Eagle medium (DMEM) (Gibco, 11995065) with 10% FBS, 2 mM l-glutamine, and 1% penicillin/streptomycin. All cell lines were maintained in a humidified incubator (37°C, 5% CO_2_) and checked regularly for mycoplasma contamination.

### Generating paired sgRNA-target site library in cell lines

Lentivirus for the sgRNA-target site library was produced as follows. 5 × 10^5^ HEK293T cells were plated in all wells of a six-well plate in 2 ml of DMEM and incubated overnight. Each well was transfected with 750 ng plasmid library, 750 ng of third-generation packaging mix [equimolar mix of pMD2.G (Addgene, #12259), psPAX2 (Addgene, #12260), and pMDLg/pRRE (Addgene, #12251)], and 10 μl polyethylenimine (PEI, Polysciences Inc., 239661, 1 mg/ml). Twenty-four hours after transfection, 3 ml of DMEM is added to each well. Lentivirus was harvested 72 h after transfection and filtered through a 0.45 μm syringe filter to remove debris. The virus from each well was pooled, resulting in ∼30 ml of virus. 10 ml of virus at three dilutions (1:2, 1:5, and 1:10) were used to infect 10^6^ wild-type K562 cells for 2 h at 1000 × *g* at 33°C in duplicate. Polybrene (Sigma–Aldrich, H92685G, 8 μg/ml) was added to the viral media. After centrifugation, cells were resuspended in regular culture media and grown for 3 days. The transduction efficiency was evaluated for each dilution via flow cytometry of mCherry percentage. We chose the 1:5 dilution of lentivirus, which had a transduction efficiency of ∼10%, which suggests that most cells contained only a single sgRNA-target site integrated. The infected cells were selected with puromycin (Sigma–Aldrich, P8833, 1 μg/ml) for 5 days until greater than 90% of the cells were mCherry positive (mCherry+). After puromycin selection, the cells were bottlenecked to reduce the complexity of the sgRNA-target site library. 10^5^ mCherry+ K562 cells (5000× coverage) were plated and expanded to grow until the introduction of base editors.

### Induction of base editing in library cells

For electroporated samples, 5 × 10^6^ bottlenecked K562 cells were centrifuged at 500 × *g* for 5 min. Cells were resuspended in 100 μl of electroporation buffer. The electroporation buffer consisted of 20 μl of Buffer I [0.2 g ATP disodium salt (Sigma–Aldrich, A6419-10G) and 0.12 g magnesium chloride hexahydrate (Fisher Scientific, BP214-500) in 1 ml] and 1 ml of Buffer II [0.06 g potassium dihydrogen phosphate (Sigma–Aldrich, P5655-100G), 0.06 g sodium bicarbonate (Sigma–Aldrich, S5761-500G), and 0.02 g glucose (Sigma–Aldrich, G7021-100G) in 50 ml at pH 7.4]. 1 μg of each plasmid being electroporated was added to the cell mixture and moved to a 2 mm cuvette. Cells were electroporated on a Lonza Nucleofector 2b using program T-016. Cells were rescued in 3 ml of warm media and allowed to rest for 24 h. Afterward, cells were expanded and maintained in log growth. Three days after electroporation, cells were sorted on a BD FACSAria II to collect the desired fluorescent cells (tagBFP positive or tagBFP/GFP positive). The number of cells collected varied between 3 × 10^5^ and 5 × 10^5^ cells. Sorted cells were grown for an additional 6 days, after which cells were collected for sequencing. For the ABE7.10;DivA-BE;Sequential sample, a second round of electroporation was performed on the ABE7.10 edited cells.

For lentivirally transduced samples, lentivirus was produced using the same protocol described for the sgRNA-target site library. 2 ml of lentivirus per plasmid were used to transduce 3 × 10^5^ bottlenecked K562 cells. Polybrene (8 μg/ml) was added to the virus-cell mixture, and the cells were centrifuged at 1000 × *g* for 2 h at 33°C. After centrifugation, the viral media was removed and replaced with fresh RPMI growth media. Cells were grown for 2 days before adding blasticidin (Research Products International, B12200, 10 μg/ml) and/or hygromycin B (Thermo Fisher, 10687010, 200 μg/ml). Cells were selected for 6 days before collection for sequencing. Bottlenecked K562 cells not transduced with editors were treated with each antibiotic in parallel to verify that the selection was complete.

### Preparation and sequencing of sgRNA-target site amplicon libraries

Genomic DNA was extracted from 3 × 10^6^ cells using QiaAmp DNA Blood Mini Kits (Qiagen, 51104) following the manufacturer’s instructions. The sgRNA-target site-UMI cassette was amplified using PCR with primers listed in Supplementary Table S2. These primers added Illumina adapters and sample barcodes. 2 × 100 μl reactions were set up for each sample. Each 100 μl reaction contained 5 μg of genomic DNA, 1 μl of 100 μM forward primer mix (oGH840.1-6), 1 μl of 100 μM reverse barcoded primer, and 50 μl of NEBNext 2× Master Mix (NEB, M0541S). oGH840.1-6 are six oligonucleotides with variable lengths to stagger and phase the sequencing of a common sequence in the target site amplicon. Samples were placed in a thermocycler and heated to 98°C for 3 min, followed by 25 cycles of 98°C for 30 s, 59°C for 30 s, and 72°C for 4 min. PCRs were pooled, and 50 μl of this mixture was analyzed on a 2% TBE agarose gel. A band at 430 bp was excised and purified with the QIAquick Gel Extraction Kit. Libraries were quantified with a Qubit dsDNA HS Kit (Thermo Fisher, Q33231) and pooled evenly except for Parent samples, which were loaded at 2× compared to other samples. PhiX control (Illumina, FC-110-3001) was spiked into the mixture of prepared samples at 30% and sequenced on an Illumina NextSeq 550 using a paired-end read of 139 cycles with the standard Read 1 primer, a 6-cycle Index 1 read with a custom primer (oGH778), and a 21-cycle read with a custom Read 2 primer (oGH779). Parent samples had 5–8 million reads, and samples with editors had 1.5–5 million reads.

### Processing of sequencing data files

Sequencing reads were demultiplexed using bcl2fastq. A bowtie (version 1.3.1) [10] (sgRNAs) and bowtie2 (version 2.4.5) [11] (target sites) indices were generated using “make_fa_sgRNA_Indices.py” and “make_fa_TargetSite_Indices_b2.py,” respectively. Common sequences were removed, and UMIs were extracted from reads using “BEChar_extract_trim.py.” This script uses umi_tools (version 1.1.6) [12] to extract the 7-bp UMI from Read 1. The UMI is appended to each Read_ID by umi_tools. Cutadapt (version 4.9) [13] removed a common primer sequence used to amplify the region from Read 1 after UMI extraction (target site) and the last base from Read 2 (sgRNA).

The fastq files from the Parent samples were aligned, and sgRNA-UMI-triplets to be included in the analysis were generated using “Define_whitelist.py.” The sgRNA alignment was done with bowtie, allowing one mismatch in the -v mode. We aligned the target site using bowtie2 in the $\text{--}$local mode. The resulting sam files were converted to bam files using samtools (version 0.1.19) [14]. We compared four read-filtering pipelines (see Supplementary Text S1). The final processing pipeline contained three read-filtering steps. In the first step, reads that failed to align to any target site were removed. Second, reads with improperly paired target site and sgRNA were removed. For the last filtering step, umi_tools grouped reads into sgRNA-target site-UMI triplets. We tabulated a list of triplets where >90% of their reads were free of insertions/deletions in the Parent sample for each replicate. This list of acceptable sgRNA-UMI-triplets was used to filter the fastqs for all samples in downstream steps. After filtering, there were >180 (90%) sgRNA-target site pairs with more than five acceptable UMIs.

### Generating allele table for Transient and Integrated samples

Fastqs generated after UMI extraction and truncation for all Transient and Integrated samples were processed using “UMI_Filter.py.” This script filters each fastq file by retaining reads that match an sgRNA-target site-UMI triplet found in our list of acceptable triplets from the Parent sample from that replicate. The subsequent fastqs were processed using CRISPResso2 (version 2.2.11) [15] using the CRISPRessoPooled command with the following settings: $\text{--}$exclude_bp_from_left 5 $\text{--}$exclude_bp_from_right 0 $\text{--}$min_reads_to_use_region 10 $\text{--}$base_editor_output $\text{--}$suppress_plots -w 0 $\text{--}$write_detailed_allele_table. We combined all of these target sites and editors into a single allele table using “CreateAlleleTables_General.py” followed by normalization of the tables using “NormalizeTables_Cutoff.py.” In the normalization script, alleles with only one read were removed as these alleles are more likely to contain mutations introduced by sequencing error, and the frequency of each allele was recalculated after removing these alleles. The analysis was applied to the Transient and Integrated samples separately, and allele tables were generated for each dataset.

Normalized allele and base substitution frequencies relative to the Parent sample were generated by “MakeAllTables_Full.r” (Transient) and “MakeAllTables_Full_Lenti.r” (Integrated). We calculated the normalized frequency of each allele detected by subtracting the frequency of the same allele in the Parent sample. For alleles not detected in the Parent sample, we set the Parent allele frequency to 0. Any negative frequencies after the subtraction of the Parent allele frequency were set to 0. If an allele was only detected in one replicate, the normalized frequency in the other replicate was set to 0. The mean editing efficiency for each allele was calculated using the normalized frequency from both replicates. This normalized frequency table was used to create a base substitution frequency table by separating each base substitution observed in a mutated allele. This separation produces a row in the table for each substitution containing its position in the allele and the normalized frequency of the allele from which the substitution is derived.

### Analysis of base editing and indel efficiency

Editing efficiency for each target site was calculated by summing the normalized allele frequency of all modified alleles with a frequency above 0.5% for that target site. Target sites with fewer than 200 reads across both replicates were removed from the analysis. For indel editing efficiency, the normalized allele frequency table was limited to alleles that had n_inserted or n_deleted >0. These two columns represent the number of inserted or deleted bases, as calculated by CRISPResso2. For the calculation of editing efficiency for alleles with CompC>N or C>N edits, the sequences were reverse-complemented for target sites where the sgRNA targeted the non-transcribed strand (bottom), converting these target sites to match those with a transcribed strand (top) targeting sgRNA. An additional column was calculated to specify whether the allele contained CompC>N, C>N, or both types of edits. C>N editing efficiency was determined using alleles with C>N or both types of edits, while CompC>N editing efficiency was computed using alleles with CompC>N or both types of edits. Editing efficiencies inside the −18 to −13 bp windows were calculated by only considering alleles that contained an edited base within −18 to −13 bp. Alleles containing edits outside the −18 to −13 bp window were used to calculate editing efficiency outside the window.

### Analysis of editing window and base substitution spectrum or purity

The base substitution frequency table was used in this analysis. The reference base and position relative to the PAM were calculated for each base substitution. The position was calculated after reverse complementing target sites with sgRNAs targeting the non-transcribed strand. Negative positions indicate that the sgRNA is upstream of the PAM. Aggregate Editing was calculated by summing the base substitution frequency at each base position for edited alleles at a frequency above 0.5%. This sum was divided by 100 to give the final Aggregate Editing value. To define whether a base position was edited, we calculated a cutoff (*μ*+ 2*σ*) based on the mean (*μ*) and standard deviation (*σ*) of Aggregate Editing at each position in the dCas9;MS2-AIDDead samples. The *μ*+ 2*σ* cutoffs were 0.189 for Transient and 0.211 for Integrated delivery systems. Therefore, 0.25 was used as a cutoff to call a base as edited for CRISPR-X (dCas9;MS2-AID*) samples, where the target sites were separated based on whether the sgRNA targeted the transcribed or non-transcribed strand of the target site. We used a 0.5 cutoff to call a base edited for editors where the deaminase was directly fused to d/n/n’Cas9 because all target sites for sgRNAs targeting either strand were combined for the analysis. The editing window was reported as the left- (least, including negative values) and right-most bases that exhibited editing above the cutoff. To calculate the editing spectrum of the base editors, we calculated the Aggregate Editing for all edits with the same reference base rather than an individual base position.

### dCas9 expression analysis using intracellular staining

Each condition was performed in three replicates with wild-type K562 cells. Lentiviral transduction and electroporation conditions were the same as the editing assay. For the electroporated samples, 3 days after electroporation, 4 × 10^6^ cells were collected from each replicate. For the transduced samples, 4 × 10^6^ cells were collected from each replicate 6 days after infection. Cells were fixed in Fix Buffer I (BD Biosciences, 557870) at 37°C for 15 min and washed with phosphate buffered saline with 10% FBS. Cells were permeabilized with Perm Buffer III (BD Biosciences, 558050) on ice for 30 min and washed again. They were incubated with primary anti-Cas9 (clone 7A9-3A3) antibody (Thermo Fisher, 61577, 1:1000 dilution) diluted in wash buffer at room temperature for 1 h. They were then washed and incubated with anti-mouse secondary antibody conjugated to Alexa647 (Thermo Fisher, A-21235, 1:1000) diluted in wash buffer at 4°C for 1 h in the dark. After incubation, cells were washed, resuspended in wash buffer, and measured via flow cytometry. For each sample, dCas9-positive and dCas9-negative cells were determined based on an uninfected/no electroporation control. The relative median dCas9 expression was calculated by normalizing the median dCas9 levels for dCas9-positive cells by the median dCas9 levels for the dCas9-negative cells.

### Measuring the distance between dCas9 termini and DNA bound by base editor

The 6vpc [16] structure was loaded into PyMOL. We calculated the distance between the following atoms in the structure. For the N- and C-termini of dCas9, we selected the ɑ-carbon of the first and last residue in the dCas9 structure, respectively. To represent the base with CompC>N editing, we selected the end of the nitrogenous base away from the phosphate backbone for the nucleotide at position −24 from the PAM on the target strand. The distance command in PyMOL was used to calculate the distance between these positions.

### Motif enrichment score at edited cytidines or guanines

For each allele with a frequency above 0.5%, we extracted the reference sequence of the 7-mer centered on the edited base (−3 to 3 position). We produced the aggregate logo by summing each of these 7-mers at each relative position, weighted by the frequency of the edited allele. The enrichment scores (ESs) were calculated using a log-likelihood ratio based on the probability of the motif among the edited cytidines/guanines compared to a background distribution based on the 52% G/C content, which was used to generate the surrounding sequence. For example, in the WRC motif, the observed probability of the motif (P_observed_) = (frequency of A or T at position −2) × (frequency of G or A at position −1). The background probability (P_background_) is the same calculation based on the 52% GC content, which would be 0.48 × 0.5 = 0.24. The ES = log_2_ (P_observed_/P_background_). A positive ES represents enrichment for the motif, while a negative value suggests the motif is depleted among the edited population.

### Quantifying base editor-induced diversification

The number of generated alleles was calculated considering alleles with a normalized frequency greater than 0.5%, excluding the unmodified reference allele. For each target site, the entropy was calculated as the sum of −*P* × ln(*P*), where *P* is the allele frequency for alleles with a frequency greater than 0.5%. This entropy calculation included the unmodified reference sequence as one of the alleles.

### Analysis of the number of edits per allele

We considered alleles with a normalized frequency above 0.5% and at least one base substitution in this calculation. The number of substitutions per allele was determined using the n_mutated field generated by CRISPResso2. The allele frequency for all such alleles was summed and grouped based on the number of mutations detected in the allele. This sum was normalized by the total editing frequency for each editor, and the proportion of the total edits for each editor was graphed.

### Quantification and statistical analysis

The graphs included in this study were generated using ggplot2 [17] or GraphPad PRISM. The logo graphs were plotted using ggseqlogo [18]. Statistical analysis was performed using PRISM. Statistical analysis was two-sided (when applicable). Additional statistical test details are included in the figure legends.

## Results

### Design, construction, and analysis of paired sgRNA-target site library

**Figure 1.**
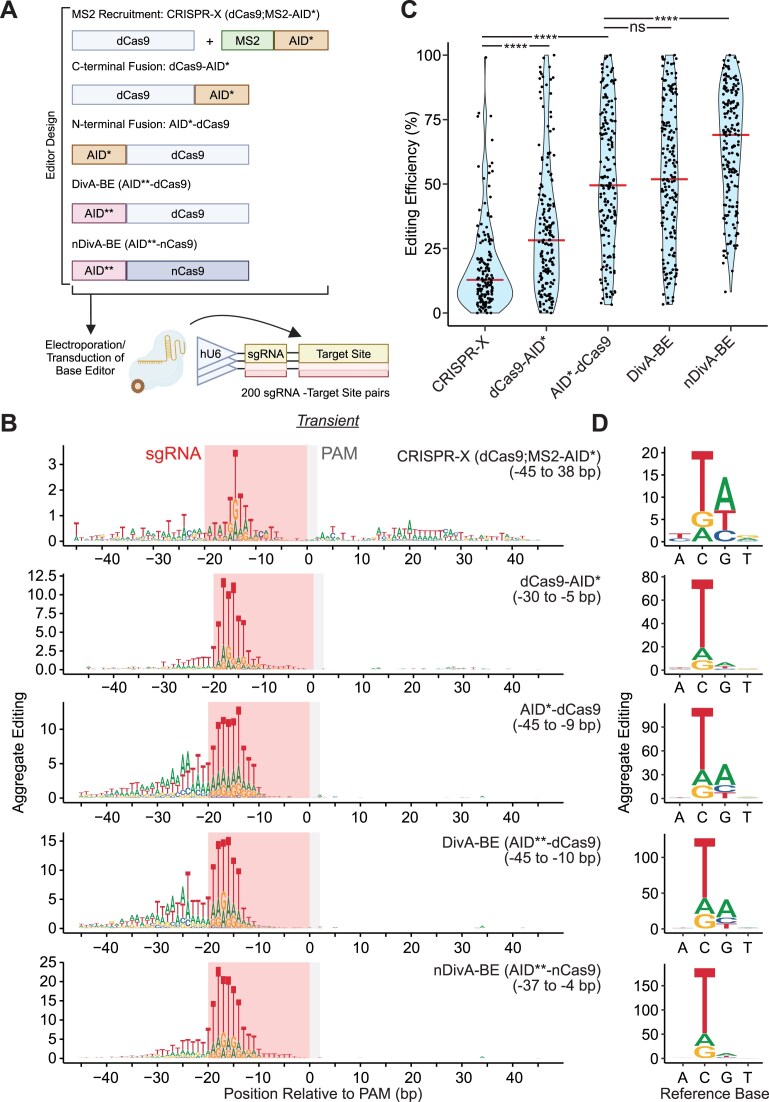
DivA-BE and AID variants fused to the N-terminus of dCas9 exhibited higher editing efficiency than C-terminal fusions or CRISPR-X. (**A**) Schematic for characterization of editing for 200 sgRNA-target site pairs. Base editors are introduced either via electroporation or lentiviral transduction to K562 cells containing a library of sgRNA-target site pairs. Schematics for several editors used in the study are shown. Additional editor schematics are in Supplementary Fig. S1C. Created in BioRender. Hess, G. (2025) https://BioRender.com/bmdatwo. (**B**) Aggregate Editing logos of base substitutions produced by Transient CRISPR-X (dCas9;MS2-AID*), dCas9-AID*, AID*-dCas9, DivA-BE (AID**-dCas9), and nDivA-BE (AID**-nCas9) base editors at each base position relative to the PAM. The bases shown indicate the resulting base substitution. The red-shaded boxes indicate the position of the protospacer sequence for sgRNAs. The gray-shaded boxes indicate the position of the NGG PAM. The left and right ends of editing windows relative to the PAM are indicated in parentheses. (**C**) Violin plots for editing efficiency of Transient CRISPR-X, dCas9-AID*, AID*-dCas9, DivA-BE, and nDivA-BE base editors. The red horizontal bars indicate the median editing efficiency. (Kruskal–Wallis test; ****P < .0001, ns = not significant). (**D**) Aggregate Editing plot of editing spectrum of base substitutions generated by Transient CRISPR-X, dCas9-AID*, AID*-dCas9, DivA-BE, and nDivA-BE base editors.

To characterize the editing outcomes for genome editors for multiple target sites in parallel, we designed a lentiviral cassette that expressed an sgRNA next to a synthetic target site targeted by the sgRNA (Fig. 1A). The sgRNA stem-loop contained two MS2 RNA hairpins [19] that can recruit cytidine deaminases fused to the MS2 coat protein [6]. Oligonucleotides encoding sgRNA-target site pairs can be inserted into this cassette and delivered to mammalian cells. After adding a Cas9-based editor, the integrated cassette can be sequenced to determine the range of edits produced across all sgRNA-target site pairs for a population of cells.

We designed 200 sgRNA-synthetic target site pairs to profile the editing activity of base editors in parallel (Supplementary Fig. S1A). sgRNAs were selected from non-targeting controls in a human genome-wide CRISPR knockout library [9]. These controls with no binding sites in the human genome avoid any confounding growth effects from editing elsewhere in the genome. We reverse-complemented half of the resulting target site sequences to facilitate analysis of whether editing patterns were altered by which strand the sgRNA targeted. Ten negative control sgRNA-target site pairs were included, which should not be edited in the presence of a genome editor. During plasmid library construction, we inserted a 7-bp UMI to distinguish between oligonucleotide synthesis errors and base editor mutations in the downstream analysis to improve the detection of editing [20]. PCR amplification of the resulting lentiviral cassette to attach sequencing adapters produces an amplicon compatible with paired-end high-throughput sequencing (Supplementary Fig. S1B).

The sgRNA-target site library was transduced into K562s, followed by selection with puromycin. To utilize the UMI to detect pre-existing mutations, we needed to determine the identity of all sgRNA-target site-UMI triplets in our cell population before editing was induced. The sequencing required to characterize all of the triplets in the infected cells in each experiment is not feasible. Doing this would require >10^8^ (10× depth for each of the ∼10^7^ transformed colonies) aligned reads for each replicate, which does not account for additional reads needed to identify mutations introduced during lentiviral production/transduction or reads lost to recombination during sequencing library preparation. To address this limitation, we bottlenecked the population to limit the diversity of sgRNA-target site-UMI triplets. The bottlenecked cells were expanded, and base editors were delivered transiently via electroporation (Transient) or integrated through lentiviral transduction (Integrated) (Supplementary Table S3, Fig. 1A, and Supplementary Fig. S1C). Multiple variants of the cytidine deaminase AID are used in this study, such as the catalytically inactive AIDDead. AID* was previously referred to as AID*Δ, which contains mutations (K10E, T82I, and E156G) that increase deaminase activity [21]. The last three residues were removed to break the nuclear export signal (NES) and further improve activity. All AID variants in this study, including AIDDead, had no functional NES, so we dropped the Δ to simplify notation. For Transient delivery, the expression vectors included a fluorescent marker (GFP or tagBFP), which was used to enrich transfected cells via FACS. For Integrated delivery, expression vectors contained antibiotic resistance markers (blasticidin or hygromycin) used to enrich transduced cells. After sorting or selection, genomic DNA was extracted from the uninfected parent cells and edited samples. We amplified and sequenced the sgRNA-target site cassette. The bottlenecking step, introduction of editors, and sequencing were performed in duplicate for all samples in the study.

We developed an analysis pipeline that robustly detected base editing in the Transient and Integrated samples (Supplementary Fig. S2A–D and Supplementary Text S2). We compared the median editing efficiency for eight editors delivered using both systems. We observed an increase in median editing efficiency for Transient editors, finding a modest correlation (Pearson = 0.518, Supplementary Fig. S2E) between the allele frequencies across delivery systems. This correlation suggests that although some allele frequencies are related, there are alleles whose frequencies were altered by the delivery method.

### Profiling by sgRNA-target site library captured the asymmetric editing pattern of CRISPR-X

To further validate our analysis pipeline, we tested whether our system could capture a known editing window of DCBEs. Previously, we defined the editing window of CRISPR-X (dCas9;MS2-AID*) [6] as −50 to 50 bp relative to the PAM. This window was dependent on the direction of transcription in the region. In our profiling assay, this dependency would result in different editing windows for sgRNAs that target the transcribed top strand compared to those that target the non-transcribed bottom strand. We observed lower editing for the sgRNAs that target the transcribed strand, which we attribute to the fact that the edits generated by these sgRNAs occur later in the Illumina sequencing read. These later base pair positions have higher sequencing error rates, which leads to higher background mutagenesis frequencies that are subtracted during analysis.

To determine the editing window for CRISPR-X, we calculated Aggregate Editing, the sum of edited allele frequencies at each base position across all target sites in the CRISPR-X Transient and Integrated samples (Supplementary Fig. S2F). We divided the target sites based on whether the sgRNA targeted the transcribed or non-transcribed strand to test whether the editing windows differed between these two target site sets for CRISPR-X. We used a cutoff for calling a base as edited based on the dCas9;MS2-AIDDead sample and defined the editing window by the left- and right-most bases with Aggregate Editing above this cutoff. We observed editing windows that extended on both sides of the PAM (Supplementary Fig. S2F) for the Transient and Integrated CRISPR-X samples. However, both observed windows are narrower than the previously defined −50 to 50 bp window. This reduction could be due to a few reasons. First, we could not detect mutations in the −45 to −50 and 45 to 50 bp regions as these overlap primers used for sequencing preparation. Second, the editing window does not suggest that editing cannot occur outside the window, but the window indicates regions consistently edited across many sgRNA-target site pairs. Given the −50 to 50 bp window was derived from the editing pattern of two sgRNAs, our results may more accurately reflect areas of consistent editing for CRISPR-X.

We observed the expected asymmetric editing pattern of CRISPR-X for sgRNAs targeting the transcribed and non-transcribed strands (Supplementary Fig. S2F). For target sites where the sgRNA targets the transcribed strand, both delivery methods had an extended window (>70 bp), while sgRNAs matching the non-transcribed strand had a narrower window (<45 bp). These results demonstrate that our profiling assay can capture the editing patterns of Cas9-mediated base editors.

### AID* fused to the N-terminus of dCas9 had higher editing efficiency than C-terminal fusions and CRISPR-X

Multiple methods of recruiting the deaminase have been used in Cas9-mediated base editor systems [22–26]. Yet, the effect of these recruitment methods on editing properties has not been studied over many target sequences. Thus, we analyzed the editing patterns for DCBEs that recruit AID* via an MS2 RNA aptamer system (CRISPR-X, dCas9;MS2-AID*), direct fusion to the C-terminus of dCas9 (dCas9-AID*), and a fusion to the N-terminus of dCas9 (AID*-dCas9) delivered transiently (Fig. 1B and C). We observed a symmetric editing pattern for both direct fusions (Fig. 1B), as all editing was detected upstream of the PAM. We found a reduced editing window for both fusions. Despite the reduced window, we saw an increase in median editing efficiency (Fig. 1C) for AID*-dCas9 (3.88-fold) and dCas9-AID* (2.20-fold) compared to CRISPR-X. The increased editing efficiency for AID*-dCas9 is in line with previous studies of N-terminal fusions of AID* [25].

For some mutational scanning assays, base editors are expressed constitutively after lentiviral transduction. Therefore, we investigated whether the method of AID* recruitment affected editing similarly for editors delivered after lentiviral integration (Supplementary Fig. S3A and B). We observed an increase in editing efficiency for CRISPR-X Integrated compared to Transient. The dCas9-AID* was the least efficient editor of the three Integrated editors, and we did not identify any positions with Aggregate Editing above the edited threshold. dCas9-AID* displayed a higher editing efficiency across all target sites when compared to the dCas9;MS2-AIDDead sample (*P* = 0.0216, Mann–Whitney test), suggesting dCas9-AID* was capable of editing alleles, albeit at low efficiency. AID*-dCas9 was the most potent editor of the three recruitment methods tested, with a similar editing window in both delivery systems. These results show that the recruitment method of AID* influences the editing efficiency and target window in a delivery-dependent way.

The observed differences in editing efficiency between the recruitment and delivery methods could be due to dCas9 expression level variations. To determine whether this was the case, we compared dCas9 levels using intracellular staining for dCas9, dCas9-AID*, and AID*-dCas9 expression vectors used in the Integrated and Transient datasets (Supplementary Fig. S3C). Transient delivery used a tagBFP vector, and the Integrated system used a vector containing a blasticidin resistance (BlasticidinR) cassette. Both sets of plasmids were delivered by both delivery methods for this assay to determine whether the plasmids differentially expressed dCas9. We used dCas9 as a proxy for CRISPR-X because dCas9 is required to target the editing site, and thus, its expression level is the limiting factor in editing with the caveat that sgRNA levels could vary with MS2 aptamers, potentially impacting editing efficiency. For both the Integrated and Transient delivery methods, dCas9 expression for dCas9-AID* and AID*-dCas9 was similar, yet AID*-dCas9 had higher editing efficiency (Fig. 1C and Supplementary Fig. S3B). Regardless of the delivery method, dCas9 had higher expression levels than the fusion vectors, though this did not result in CRISPR-X being the most potent editor. Therefore, changes in editing efficiency between recruitment methods were not explained by dCas9 expression. The only difference we observed was an increase in expression for the transiently delivered dCas9-tagBFP compared to its dCas9-BlasticidinR counterpart. Surprisingly, we observed similar dCas9-AID* expression levels across delivery methods despite the differences in editing efficiency. While we did not find a change in dCas9-AID* expression at time points in this experiment, this does not rule out that expression levels could change over the editing timeframe. The Integrated measurements were taken at the maximum expression state 6 days post-infection. In contrast, the Transient measurement was taken 3 days after electroporation, which coincided with peak expression based on tagBFP levels, but others have found that higher expression could occur at an earlier time after 10 h [27]. Thus, in the Transient group, higher dCas9 expression levels at an earlier time point could explain the observed editing efficiency differences between the Transient and Integrated dCas9-AID* samples. Overall, dCas9 expression levels did not correlate with changes in editing efficiency across recruitment or delivery methods and do not explain the observed changes in editing efficiency.

An advantage of DCBEs is that they produce C>N mutations rather than only C>T, although the distribution of these edits has only been studied in low-throughput for a few target sites. We analyzed the editing spectrum of substitutions made by each base editor recruitment method **(**Fig. 1D and Supplementary Fig. S3D). C>T was the most common substitution for all editors in this analysis. For Transient CRISPR-X, we observed a 67:16:18 distribution of C>T:A:G substitutions. Both AID*-dCas9 and dCas9-AID* showed a similar distribution with a slightly increased preference for C>T mutations. For Integrated editors, we observed a similar preference for C>T edits, although C>G edits comprised a higher proportion (>30%) of the editing spectrum for Integrated CRISPR-X and dCas9-AID*. These results show that AID*-based DCBEs can effectively make C>N edits with a preference for C>T edits in the tested recruitment and delivery methods.

### DivA-BE and nDivA-BE, fusions of truncated hyperactive AID variant (AID**) to d/nCas9, increased editing efficiency

The AID*-dCas9 had the highest editing efficiency among the recruitment methods for either Transient or Integrated delivery. However, we sought to improve editing efficiency further. We tested two additional constructs using a more potent hyperactive mutant AID** [21], which contained three additional mutations (S66T, L104I, and K160E) and truncated an additional 15 residues from the C-terminus of AID. To determine whether this deaminase could further improve editing efficiency, we fused AID** to dCas9, which we named DivA-BE. Additionally, we fused AID** to nCas9 (nDivA-BE), which should increase base editing efficiency by biasing repair to use the deaminated non-target strand as the template in repair [28]. For Transient delivery, we observed a small, nonsignificant increase in median editing efficiency for DivA-BE compared to AID*-dCas9 (Fig. 1C). However, nDivA-BE exhibited an increase in editing efficiency over both dCas9 editors. In the Integrated delivery samples, we observed a relative increase in editing efficiency (Supplementary Fig. S3B), although DivA-BE had a more substantial editing efficiency increase compared to AID*-dCas9.

The editing window for DivA-BE was similar to AID*-dCas9 (Fig. 1B and Supplementary Fig. S3A). Despite the increased editing efficiency, nDivA-BE had a shifted editing window toward the PAM for both Transient and Integrated delivery and edited positions −45 to −38 less efficiently than the dCas9 fusions. Neither DivA-BE/nDivA-BE editors exhibited a change in C>T:A:G editing spectrum compared to AID*-dCas9, with 64%–72% of cytidine edits resulting in C>T substitutions (Fig. 1D and Supplementary Fig. S3D). These results show that DivA-BE and nDivA-BE have improved editing efficiency compared to AID*-dCas9.

### DivA-BE and AID*-dCas9 produced C>N and CompC>N mutations with shifted targeting windows

**Figure 2.**
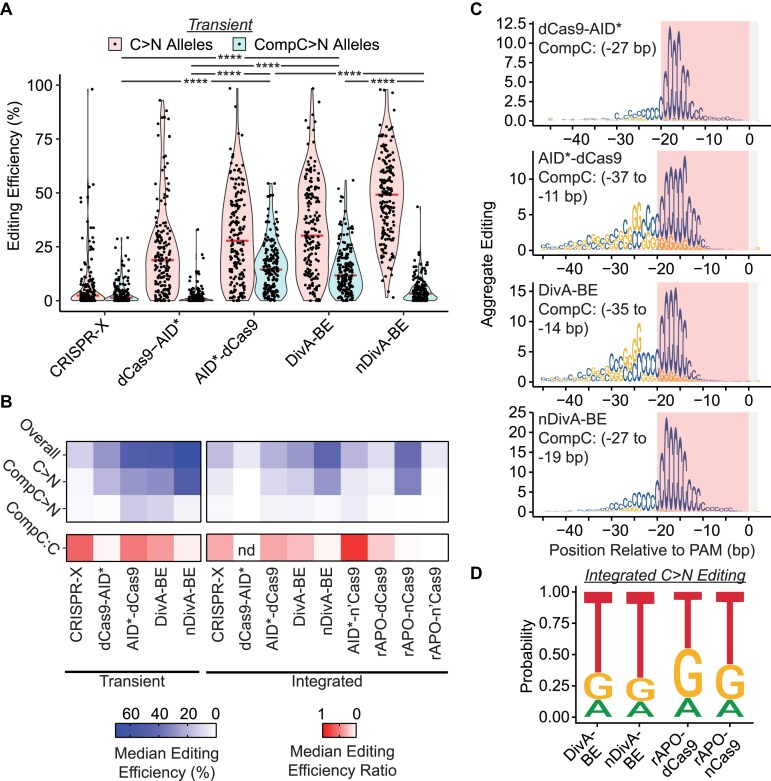
DivA-BE generated CompC>N mutations with a shifted window from C>N mutations. (**A**) Violin plots for editing efficiency of Transient CRISPR-X, dCas9-AID*, AID*-dCas9, DivA-BE, and nDivA-BE base editors. Red violin plots represent the editing of alleles containing C>N mutations, and blue plots indicate efficiency for G>N mutations. Red horizontal bars indicate the median editing efficiency. (Kruskal–Wallis test; ****P < .0001). (**B**) Heat map of median editing efficiencies (blue) and the ratio of median CompC>N and C>N editing efficiency (red; CompC:C) for Transient and Integrated editors. (nd - ratio not determined since median C>N editing was 0). (**C**) Aggregate Editing logos of reference base substitutions produced by Transient dCas9-AID*, AID*-dCas9, DivA-BE, and nDivA-BE base editors at each base position relative to the PAM. The bases shown indicate the reference base that was altered. The red-shaded boxes indicate the position of the protospacer sequence for sgRNAs. The gray-shaded boxes indicate the position of the NGG PAM. The CompC editing window is indicated on the graphs. (**D**) Normalized editing spectra for C>N editing in DivA-BE, nDivA-BE, rAPOBEC1-dCas9 (rAPO-dCas9), and rAPOBEC1-nCas9 (rAPO-nCas9) base editors for Integrated delivery.

In addition to the expected C>N mutations, we observed the presence of G>N mutations for AID*-dCas9 and DivA-BE samples (Fig. 1D and Supplementary Fig. S3D). The most straightforward explanation of these G>N mutations was the deamination of cytidines on the target strand. If these G>N edits were produced by AID-mediated deamination, we hypothesized that the distribution of edits (G>A:T:C) would be similar to those of C>T:A:G, since the same mechanism would repair them. We observed a similar distribution for G>A:T:C and C>T:A:G edits for both AID*-dCas9 and DivA-BE with both delivery systems (Supplementary Fig. S4A), so we referred to these G>N edits as Complementary C>N (CompC>N) editing. We analyzed the editing efficiency of alleles that contain either C>N or CompC>N mutations (Fig. 2A and Supplementary Fig. S4B) to determine whether the CompC>N mutations were present at a few target sites or were common across most editing sites. For both Transient editors and Integrated editors, we observed median editing efficiency greater than 10% for CompC>N alleles in the AID*-dCas9/DivA-BE samples, suggesting CompC>N editing occurred at most target sites tested in our assay. Furthermore, we noted that CRISPR-X, dCas9-AID*, and nDivA-BE samples had lower CompC>N editing efficiencies. This decrease in CompC>N editing could be due to reduced overall editing efficiency, so we computed the ratio of median CompC>N editing efficiency to median C>N editing efficiency (Fig. 2B). This ratio was lower in the dCas9-AID* and nDivA-BE samples, while CRISPR-X had higher relative CompC>N editing than AID*-dCas9 and DivA-BE editors. We observed a similar relative depletion of CompC>N edits for Integrated nDivA-BE. The Integrated dCas9-AID* had a median CompC>N and C>N editing of 0, so we could not compare the CompC>N:C>N ratio to other editors.

nDivA-BE and dCas9-AID* exhibited lower levels of CompC>N editing and reduced editing efficiency outside the sgRNA target sequence (positions −45 to −20) compared to the AID*-dCas9/DivA-BE fusions (Fig. 1B and Supplementary Fig. S3A). We hypothesized that these two observations may be related. Therefore, we analyzed the reference bases being mutated and their positions for the Transient-delivered editors (Fig. 2C). We observed ∼20–25 bp CompC>N editing windows for DivA-BE and AID*-dCas9 and a narrower window for nDivA-BE. Only one base position had CompC>N editing above our cutoff for Transient dCas9-AID*. We observed a similar trend for Integrated editors (Supplementary Fig. S4C). nDivA-BE and dCas9-AID* did not have any positions with aggregate CompC>N editing above our cutoff. For all editors where CompC>N editing was detected above our cutoff, the four positions with the highest CompC>N editing were in the −27 to −24 bp window, which is shifted from the −18 to −15 window of maximum C>N editing. The shifted windows for CompC>N editing may explain the increased editing efficiency for AID*-dCas9/DivA-BE editors outside the sgRNA sequence window.

Deamination of the target strand is consistent with the depletion of CompC>N edits by fusing the nCas9, which nicks the target strand. This single-stranded DNA (ssDNA) break biases repair toward the non-target strand and disfavors repair of deaminations of the target strand [28, 29]. However, this model does not explain why dCas9-AID* has reduced CompC>N editing. We hypothesized that this may be due to the proximity of the dCas9’s termini to the CompC>N editing window. We analyzed the structure of an adenine base editor (ABE) [16] to determine the distance of the N- and C-termini of dCas9 from the −24 bp nucleotide position, a position with a high level of CompC>N editing for both AID*-dCas9 and DivA-BE (Supplementary Fig. S4D). We analyzed this structure rather than cytidine base editor structures because it contained nucleotides that extended beyond the −20 bp window to where the maximal CompC>N edits occurred. The N-terminus was closer to the −24 bp position than the C-terminus (4.45 nm versus 8.21 nm). This increase in distance supports that the deaminase fused to the C-terminus may less efficiently interact with these positions of maximal CompC>N editing. Altogether, these results suggest that base editors with AID variants fused to the N-terminus of dCas9 produce CompC>N editing via deamination of the target strand.

### Nickase activity of Cas9 and deaminase identity altered relative CompC>N editing levels

Given CompC>N edits were sensitive to nCas9, we hypothesized that introducing a Cas9 variant that nicks the non-target strand (n’Cas9) could increase the efficiency of CompC>N editing. Furthermore, we wanted to investigate whether base editors with other deaminases besides AID produced CompC>N edits. While the CompC>N editing window overlapped the editing window observed for other cytidine deaminases, AID-mediated base editors have extended editing windows compared to editors utilizing other deaminases [4], which may be necessary to facilitate CompC>N editing. To investigate these effects, we analyzed the editing patterns of DivA-BE, nDivA-BE, AID*-dCas9, and AID*-n’Cas9 delivered via integration (Supplementary Fig. S4B and C). We also analyzed the editing generated by rAPOBEC1 fused to dCas9, nCas9, and n’Cas9. We observed an increase in the ratio of CompC>N editing to C>N editing for AID*-n’Cas9 compared to AID*-dCas9 or DivA-BE (Fig. 2B). The increase in CompC>N editing efficiency was slight and not significant. This result suggests that the primary source of the relative CompC>N enrichment was n’Cas9 reducing C>N editing. For rAPOBEC1 samples, we observed low levels of CompC>N median editing (<2) for all fusions, similar to the levels observed for nDivA-BE (Supplementary Fig. S4B). When we analyzed CompC>N Aggregate Editing at each base position, we did not observe any positions above our cutoff in any of the rAPOBEC1 fusions. These results suggest that n’Cas9 can alter the relative CompC>N:C>N editing levels and that AID** makes CompC>N edits more efficiently than rAPOBEC1.

### Deaminase in the base editor altered the spectrum of base substitutions

For AID*/AID**, we found the editing spectrum to be ∼60:20:20 for C>T:A:G mutations. Previous studies have noted a difference in editing between deaminases [23, 30], but these experiments were performed in the background of a UGI, which biases the repair of the deaminated cytidines. To investigate whether deaminases affect the editing spectrum without the UGI, we compared the editing spectrum for cytidines edited by rAPOBEC1-d/nCas9 with DivA-BE/nDivA-BE samples (Fig. 2D). Both deaminases preferentially introduced C>T edits. However, both rAPOBEC1 fusions produced a higher proportion of C>G edits (>28% in both delivery systems). These results are consistent with other findings for rAPOBEC1 editors, as variants of rAPOBEC1 have been developed with enhanced C>G editing [31, 32]. Furthermore, the knockdown of the REV1 translesion polymerase, which is responsible for inserting a guanine opposite of the cytidine [33], was found to alter C>G editing in a CRISPRi screen [32]. Combined with our results, this suggests rAPOBEC1 may engage factors different from other deaminases that alter the repair of deaminated cytidines.

### Editing window and purity of C>T programmable editors were affected by deaminase identity

**Figure 3.**
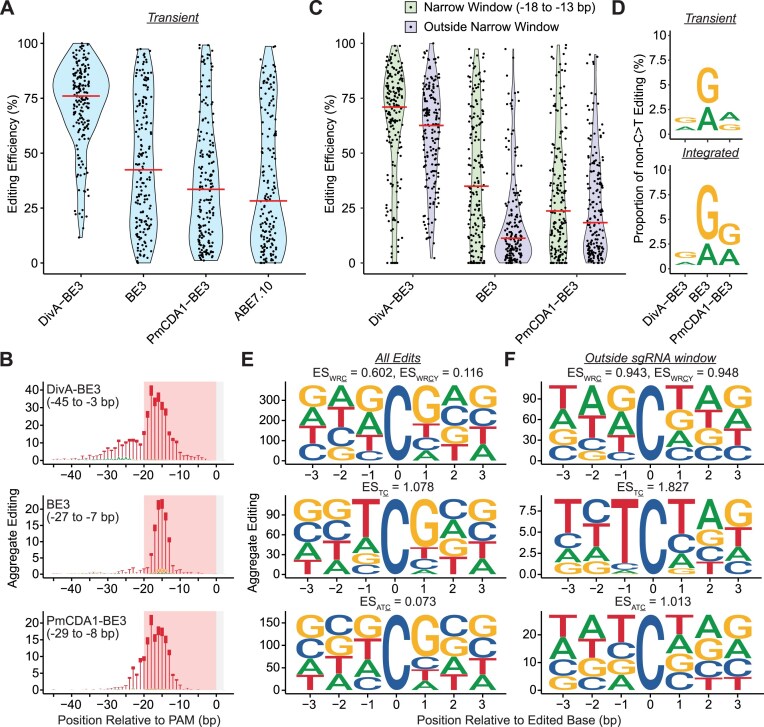
Editing window, spectra, and motif preferences for C>T editors were altered by deaminase identity. (**A**) Violin plots for editing efficiency of Transient DivA-BE3, BE3, PmCDA1-BE3, and ABE7.10 base editors. The red horizontal bars indicate the median editing efficiency. (**B**) Aggregate Editing logos of base substitutions produced by Transient DivA-BE3, BE3, and PmCDA1-BE3 base editors at each base position relative to the PAM. The bases shown indicate the resulting base substitution. The red-shaded boxes indicate the position of the protospacer sequence for sgRNAs. The gray-shaded boxes indicate the position of the NGG PAM. (**C**) Violin plots for editing efficiency of Transient DivA-BE3, BE3, and PmCDA1-BE3 base editors. Green violin plots represent the efficiency of alleles containing edits in the narrow window (−18 to −13 bp), and the purple plots indicate the efficiency of alleles containing edits outside the narrow window. The red horizontal bars indicate the median editing efficiency. (**D**) Proportion of non-C>T editing for Transient- and Integrated-delivered DivA-BE3, BE3, and PmCDA1-BE3 base editors. (**E**) Position-weight matrix for cytidines edited by Transient DivA-BE3, BE3, and PmCDA1-BE3. ESs for motifs are indicated above each graph. (**F**) Position-weight matrix for edited cytidines outside the sgRNA window (−20 to 0 bp) for Transient DivA-BE3, BE3, and PmCDA1-BE3. ESs for motifs are indicated above each graph.

Programmable C>T base editors have been used in scanning mutagenesis studies [34–36]. The abundance of the sgRNA is the readout for enrichment/depletion in these assays, but this assumes that the sgRNA produces the expected mutation with high purity. Therefore, knowing the editing windows and spectrum for programmable editors is critical for their use in such applications. Thus, we compared the editing properties for three C>T editors (DivA-BE3, BE3 (rAPOBEC1), and PmCDA1-BE3 (cytidine deaminase from sea lamprey [37, 38]) in our profiling assay (Fig. 3A and B, and Supplementary Fig. S5A and B). All these editors were in a BE3 configuration, consisting of a cytidine deaminase fused to nCas9 with a C-terminal fusion to a UGI. DivA-BE3 was the most efficient editor and had the largest editing window in both delivery systems. BE3 and PmCDA1-BE3 had similar editing windows, although BE3 had a higher median editing efficiency. Despite having similar editing windows, we noted a rapid decrease in BE3 editing efficiency beyond the −18 to −13 bp window, suggesting a narrower editing window. Therefore, we quantified the editing for the Transient and Integrated BE3 editors inside and outside the −18 to −13 bp window (Fig. 3C and Supplementary Fig. S5C). The BE3 editor had ∼3-fold more editing in the narrow window, while AID** and PmCDA1 exhibited a more modest increase (1.1–1.6-fold), suggesting BE3 has a narrower editing window than the other two deaminases. These results demonstrate how deaminase identity alters the targeting window of C>T base editors.

Another critical property of C>T editors in assays assessing the function of variants is their ability to produce C>T editors and avoid other base substitutions. Adding a UGI impairs the activity of uracil-DNA glycosylases [39] needed to initiate base excision repair, which increases the proportion of C>T edits. Nonetheless, non-C>T mutations can occur, and these low levels of non-C>T edits can be enriched through phenotypic selection, leading to the improper annotation of the functional mutation. Therefore, we analyzed the purity of the C>N edits produced by all three BE3 editors (Fig. 3D). We observed high C>T editing purity (>90%) for all editors in both delivery systems. BE3 produced the lowest purity in comparison with DivA-BE3 and PmCDA1-BE3 editors. For BE3, the impurities were most frequent within the narrow window (−18 to −13 bp), but the purity for cytidine edits at those bases was higher than that for the full editing window. This result suggests that the increased frequency of non-C>T edits was due to higher deamination activity at these positions rather than any differences in the repair of those base positions.

ABEs have also been used to assess the function of mutations [40]. Therefore, we profiled the ABE editor ABE7.10, which was delivered transiently. This editor consists of an engineered dimer of TadA, a bacterial adenosine deaminase, fused to nCas9 [41]. The deaminase converts an adenosine to an inosine, which leads to an A>G conversion during DNA replication. The median editing efficiency for the ABE7.10 was lower than that of the programmable CBEs (Fig. 3A), but it had the narrowest editing window (Supplementary Fig. S3D). The editing purity for ABE7.10 was similar to BE3 editors at >95% A>G purity. Although TadA’s primary function is deaminating adenines, we found a low frequency of cytidines being mutated at −15 to −13 bp, the three most efficiently edited positions for the ABE (Supplementary Fig. S3D). These findings are consistent with other reports that the engineered TadA enzyme can deaminate cytidines, albeit less efficiently than adenines [42]. These findings support that ABE7.10 is an efficient A>G editor, but cytidines in that window may be subject to low-frequency mutagenesis.

### Cytidine deaminase motif preferences were enhanced outside the sgRNA-targeting window

Cytidine deaminases edit cytidines in specific motifs more efficiently, and the motif preference differs between deaminases. In their native function, the deaminases may interact with other proteins to target a cytidine for deamination, but the targeting preference could be altered when the deaminase is recruited by d/nCas9. Therefore, we analyzed the motifs of the cytidines edited by the three BE3 editors (Fig. 3E and Supplementary Fig. S5E). Previous studies have found that AID preferentially edits a WRC [43] or WRCY [44, 45] motif, while the rAPOBEC1 preferentially edits a TC motif [7]. For PmCDA1, a strong preference for ATC was identified when the enzyme was overexpressed in yeast [46]. To test the preference for these motifs, we calculated a motif ES for each motif based on the observed editing. For this score, a positive value represents an enrichment for that motif, while a negative score represents depletion. For DivA-BE3, we observed positive ES_WRC_ scores >0.5 for both delivery systems. However, we found that including this extra base in the motif lowered the ES_WRCY_ score, although the scores were positive. For BE3, we observed stronger enrichment for the TC motif with ES_TC_ > 1. The PmCDA1-BE3 editor had weak enrichment for the ATC motif with ES_ATC_ < 0.25 for both delivery systems.

For all three BE3 base editors, we observed maximal editing within the −20 and 0 bp positions. We hypothesized that these positions may undergo more frequent deamination due to the proximity of the deaminase through its fusion to nCas9, independent of the deaminase’s motif preference. If this were true, we expected that edits outside this window may exhibit an enhanced motif preference, as these edits are less favored by the fusion of the deaminase to nCas9. To test this possibility, we performed the motif enrichment analysis of edited cytidines but only included edits outside the −20 to 0 bp region (Fig. 3F and Supplementary Fig. S5F). We observed an increase in ESs for the known editing motifs for all three editors, supporting that edits outside the −20 to 0 window are more affected by the motif surrounding the cytidine.

Given the enhanced motif preference for cytidines edited outside the −20 to 0 bp window, we examined whether CompC>N sites exhibited a similar enhancement of motif preference. In the analysis of AID*-dCas9/DivA-BE samples, we observed the same, although attenuated, increase in ESs for WRC/WRCY and complementary GYS/RGYS motifs for edits outside the −20 to 0 bp window (Supplementary Text S3 and Supplementary Fig. S5G).

### The introduction of a uracil glycosylase inhibitor reduced the indel efficiency of cytidine base editors

An advantage of base editors over other genome editing strategies for investigating the function of protein-coding variants is avoiding double-strand break intermediates. Bypassing this intermediate reduces the toxicity of the genome editor and avoids the production of insertions/deletions (indels), which can knock out the protein function via a frameshift rather than install the variant of interest. To assess the potential for DCBEs to introduce indels, we compared the frequency of alleles containing indels for the editors in these studies (Supplementary Fig. S6A and B). We observed low median indel editing efficiency (<10%) for all editors except for nDivA-BE. DivA-BE was among the editors with the highest indel editing efficiency, although the editor produced 1.5–3-fold fewer indels compared to nDivA-BE. The proposed mechanism for cytidine base editors producing indels is the excision of the deaminated cytidine to form an abasic site that an endonuclease can process to create a ssDNA break. The ssDNA break combined with other spontaneous nicks/ssDNA breaks or those generated by additional deamination can form double-strand breaks, which result in indels. Therefore, we hypothesized indel efficiency may be correlated with editing efficiency, as the ability of the editor to deaminate a nucleotide is required for producing base substitutions and indels. We calculated the correlation between the median indel editing efficiency and overall editing efficiency for all cytidine base editors tested in our assay (Supplementary Fig. S6C). We observed a low to moderate correlation. However, we noted that the three BE3 editors, which contain a UGI that inhibits the excision of the deaminated cytidine, exhibited low indel efficiency relative to their efficiency. We calculated the Pearson correlation, excluding the DivA-BE3, BE3, and PmCDA1-BE3 samples, and observed a high correlation between indel and editing efficiency among the other editors. These data support the model that the base excision step is critical for producing indels induced by cytidine base editors.

### Efficiency and window size affected the allelic diversity and number of edits produced by DCBEs

**Figure 4.**
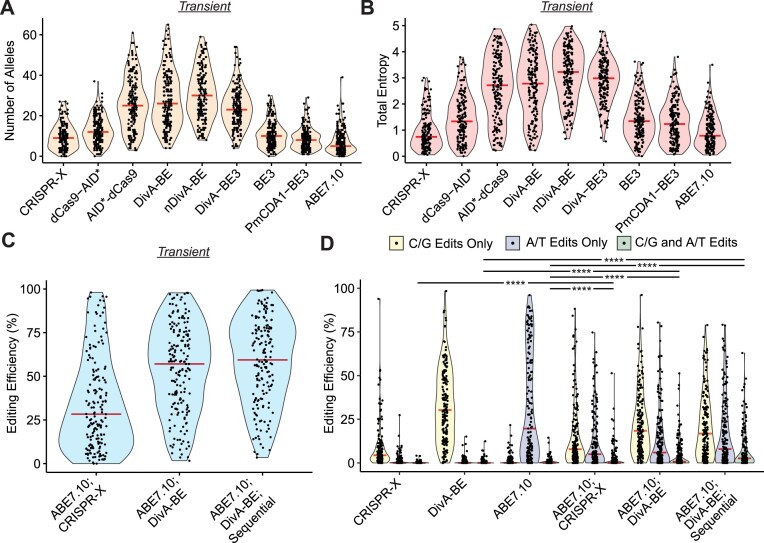
Cytidine base editors diversified target sites alone or in combination with adenine base editors. (**A**) Violin plots for the number of alleles generated by Transient base editors. Red horizontal bars indicate the median number of alleles generated. (**B**) Violin plots for the total entropy generated by Transient base editors. Red horizontal bars indicate the median entropy generated. (**C**) Violin plots for editing efficiency of combined cytidine and adenine base editors: ABE7.10;CRISPR-X, ABE7.10;DivA-BE, and ABE7.10;DivA-BE;Sequential. Red horizontal bars indicate the median editing efficiency. (**D**) Violin plots for editing efficiency of alleles containing C/G edits only, A/T edits only, or both types of edits for combinations of cytidine and adenine base editors: ABE7.10;CRISPR-X, ABE7.10;DivA-BE, and ABE7.10;DivA-BE;Sequential. The red horizontal bars indicate the median editing efficiency. (Kruskal–Wallis test; ****P < .0001).

The ability of DCBEs to generate alleles that can be interrogated for phenotypic effects is critical for their usage in scanning mutagenesis assays. We assessed the ability of the editors in the study to diversify their target sites using two metrics. The first was the number of alleles generated by a single sgRNA (Fig. 4A and Supplementary Fig. S7A). The second was the entropy generated by each sgRNA, as this measurement would consider the frequency of the generated alleles (Fig. 4B and Supplementary Fig. S7B). For both Transient and Integrated editors, nDivA-BE produced the highest median number of alleles (30, Transient; and 35, Integrated) and the highest median entropy. To determine the contribution of editing efficiency to these properties, we calculated the correlation between median editing efficiency and median entropy and number of alleles (Supplementary Fig. S7C). We observed a strong correlation between median editing efficiency and the number of alleles and entropy for each editor (Pearson_NumAlleles_ = 0.764 and Pearson_Entropy_ = 0.942). However, efficiency is not the only determinant of diversifying ability, as DivA-BE3, our most efficient editor, was not the top diversifier by either metric.

Bystander mutations, where other bases are mutated besides the desired mutation, can confound the interpretation of which mutation is functional in high-throughput base editor variant screens [47]. Therefore, we analyzed the alleles generated by the editors to determine the number of mutations on each allele (Supplementary Fig. S7D and E). For the Transient delivered editors, alleles with a single mutation had a plurality in all conditions except for nDivA-BE, DivA-BE3, and PmCDA1-BE3.

These alleles with multiple edits could be due to repeated deamination events or the processivity of the deaminase. Only CRISPR-X, BE3, and ABE7.10 had >50% of edited alleles containing a single mutation. BE3 and ABE7.10 had the narrowest editing windows of all the editors analyzed, suggesting that the processivity of the enzymes may play a critical role in controlling the number of edits on an allele. However, this is unlikely to be the sole determinant, as CRISPR-X had the largest editing window of any of the Transient delivered editors investigated. To further distinguish the effect of multiple deamination events, we performed the same analysis on the number of mutations per allele for the Integrated editors (Supplementary Fig. S7E). Given these editors were constitutively expressed, they have a greater chance of deaminating a site multiple times, as a single mutation may not disrupt Cas9 re-targeting the target site, especially if the mutation is outside the sgRNA binding site. Other than dCas9-AID*, we did not observe changes in the distribution of the alleles based on the number of mutations compared to the results for the Transient editors, suggesting that time exposure to the base editor is not a major factor in the number of edits per allele. The change for dCas9-AID* was not surprising since the Integrated sample exhibited a reduction in editing efficiency (Fig. 1C and Supplementary Fig. S3B). These results suggest that the editing window is one critical factor in determining the number of mutations per allele.

### Combinations of ABE and AID-based editors edited alleles in parallel and sequentially

Our previous analysis investigated the ability of either a CBE or ABE to work individually. Combining these two base editors in a single assay could generate additional diversity that can be interrogated for phenotypic effects. Thus, we transiently delivered ABE7.10 combined with either MS2-AID* (ABE7.10;CRISPR-X) or DivA-BE (ABE7.10;DivA-BE). Additionally, we performed sequential editing by first delivering ABE7.10 and a subsequent round of electroporation to deliver DivA-BE (ABE7.10;DivA-BE;Sequential). Both samples with DivA-BE had a higher median editing efficiency (Fig. 4C) than the ABE7.10;CRISPR-X sample. Sequential delivery of ABE7.10 and DivA-BE had the highest editing efficiency. We assessed the ability of these combined editors to diversify their target sites by calculating the number of alleles (Supplementary Fig. S7F) and entropy (Supplementary Fig. S7G) generated by each combination. We observed the highest median number of alleles and entropy for the sequential editor combination. These values were similar to those of nDivA-BE, but these were increases over DivA-BE or ABE7.10. For each editor combination, the ABE and CBE could be editing alleles independently, such that no allele would contain both a mutated cytidine and adenine. To determine whether co-editing occurred, we evaluated the frequency of alleles that contained only C/G edits, A/T edits, or both (Fig. 4D). For alleles containing both types of edits, we observed an increase in mean editing efficiency for all three combination editors, although only the combinations with DivA-BE increased the median. ABE7.10;DivA-BE;Sequential maintained the highest mean and median among the combinations tested. These results show that the combination of these editors can make multiple edits to a single allele and that sequential delivery of these editors leads to a modest increase in editing efficiency and diversification.

## Discussion

High-throughput assays using programmable and diversifying base editors have great potential for performing scanning mutagenesis in mammalian cells. These tools can identify loss- and gain-of-function mutants to uncover the molecular mechanisms for a gene of interest. Characterizing the editing properties of genome editors is critical for assessing their suitability for these assays. Our results show the value of performing these characterizations comprehensively across many targets, as the context (e.g. surrounding sequence) can better define editing properties. For example, our results defined a narrower editing window for the CRISPR-X editor, which was originally defined from only two sgRNAs. While this study marks the most comprehensive characterization of diversifying base editors to date, programmable base editors have been profiled for thousands of sgRNA-target site pairs [4, 5], enabling the application of machine learning algorithms to predict editing outcomes. Studies at this scale would be valuable for further defining or predicting the activity of diversifying base editors. However, the smaller library used here may be well suited for surveying the editing efficiency, window, and spectrum of many base editor configurations to prioritize editors with desirable characteristics for more intensive characterization with a library containing more target sites.

Our study provides insight into which editors are optimal for high-throughput scanning mutagenesis studies. There are two main approaches to using base editors to identify functional variants: tiling regions to discover mutations or installing specific variants via a programmable base editor. Based on the findings in our study, we recommend DivA-BE for variant discovery due to the increased window size (>20 bp) and diversification potential. In particular, the larger editing window allows for mutagenesis of large genomic regions with fewer sgRNAs. While nDivA-BE created the most allelic diversity (as measured by the number of alleles or entropy), this editor produced a higher frequency of alleles containing multiple mutations or indels, which may produce unwanted knockouts when editing protein-coding regions. DivA-BE may not be the ideal editor in all high-throughput diversification assays, as the larger editing window and production of CompC>N edits may make determining the causal mutation more challenging. Our data suggest that rAPOBEC1-nCas9 editor may be more desirable for diversifying narrower windows with a lower frequency of CompC>N mutations.

For installing specific C>T variants, we suggest that base editors utilizing rAPOBEC1 or other editors with narrow editing windows are preferable to AID** and PmCDA1 editors, despite DivA-BE3 being more efficient. DivA-BE3 produced a higher frequency of alleles containing more than one mutation, which makes interpreting the causal mutation difficult in these experiments. One drawback of the rAPOBEC1 editors was their lower C>T editing purity. While the C>T purity remained above 90%, these non-C>T edits, even at low frequency, can be enriched upon phenotypic selection in mutagenesis studies, perhaps misidentifying the causal mutation. Newer versions of rAPOBEC1-based editors like BE4 include two UGIs to improve C>T purity [30], but the non-C>T edits must be considered in any assay where the sgRNA identity alone is used to predict the causal mutation.

Our data suggest that some editing properties may be tunable by the recruitment method of the deaminase, as we noted that fusing AID variants to either the N- or C-termini of dCas9 reduced the editing window compared to CRISPR-X, which uses an MS2 aptamer recruitment system. This tunability of editing windows between recruitment methods may not extend beyond AID variants, as recent work showed that recruitment of rAPOBEC1 variants through an RNA aptamer system did not have an extended editing window compared to the direct fusions of rAPOBEC1 [48]. The differences in editing between the recruitment methods were not solely due to changes in editor expression. Although we observed a surprising finding for dCas9-AID* editing between delivery methods, we did not observe a correlation between the expression of AID*-based editors and their editing efficiency and windows, although delivering the editors as ribonucleoproteins may be necessary to control dosage and definitively account for expression effects.

While this study’s primary focus has been understanding how these editors would behave in mutagenesis studies of endogenous loci, our findings have relevance to therapeutic base editing applications. We observed CompC>N for DCBEs, which could produce unwanted or deleterious bystander mutations. Although we did not detect significant CompC>N editing for rAPOBEC1 and nCas9 base editors, the existence of CompC>N editing for AID*-dCas9 and DivA-BE suggests that an ssDNA substrate must be present for AID to deaminate the target strand. The ssDNA substrate may be targetable by other base editors or endogenous deaminases. Despite their low frequency, introducing such mutations should be carefully considered in therapeutic applications of base editors.

### Limitations of the study

This study defines large editing windows extending to 45 bp away from the PAM. The true windows for those editors may extend beyond this position, as we were unable to detect mutations reliably beyond the ± 45 bp window at the target site. We could not extend our analysis to these windows, as the UMI is on the 5′ end of the target site, where it is challenging to distinguish mutations from different UMIs. The 3′ end was next to the primer site needed for library amplification. Therefore, the primer may introduce any mutations detected here rather than through base editing. These limitations could be addressed in future sgRNA-target site library designs with larger target sites and incorporating longer sequencing reads.

We characterized the activity of these base editors in a single cell line. We selected K562s for this study as the cell line is commonly used in high-throughput studies and has been thoroughly characterized by the ENCODE consortium [49]. Therefore, our findings should be directly applicable to the many studies in K562s. The findings of this study may not hold in additional cell lines, as expression levels of the base editor or DNA repair activity can vary. A recent study observed a strong correlation of programmable base editing between cell lines over many sgRNAs, although there were changes in mean base editing efficiency between lines [36]. Therefore, we expect relative efficiency trends to apply in additional cellular contexts. Nonetheless, we cannot rule out critical differences in the activity of DCBEs in other cellular contexts, but extending our assay to additional cell lines should elucidate these differences and enhance our understanding of diversifying base editors and their application in mutagenesis studies.

## Supplementary Material

gkaf620_Supplemental_Files

## Data Availability

The sequencing datasets generated are deposited at the Sequencing Read Archive. Plasmids under Bioproject PRJNA1180481 (https://www.ncbi.nlm.nih.gov/bioproject/PRJNA1180481). Plasmids used in this study will be deposited at Addgene and are available upon request. The scripts used in this analysis are available on GitHub (https://github.com/HessLabUW/BECharacterization) and Zenodo (https://doi.org/10.5281/zenodo.15053238).
